# Abnormal Epigenetic Regulations in the Immunocytes of Sjögren’s Syndrome Patients and Therapeutic Potentials

**DOI:** 10.3390/cells11111767

**Published:** 2022-05-27

**Authors:** Peng Li, Mengwei Han, Xingyu Zhao, Guanqun Ren, Si Mei, Chao Zhong

**Affiliations:** 1 Beijing Key Laboratory of Tumor Systems Biology, School of Basic Medical Sciences, Institute of Systems Biomedicine, Peking University Health Science Center, 38 Xueyuan Road, Beijing 100191, China; 755368067@pku.edu.cn (P.L.); 1410305109@pku.edu.cn (M.H.); 1510305138@pku.edu.cn (X.Z.); rgq_hsc@pku.edu.cn (G.R.); 1810305114@pku.edu.cn (S.M.); 2 NHC Key Laboratory of Medical Immunology, Peking University, Beijing 100191, China; 3 Key Laboratory of Molecular Immunology, Chinese Academy of Medical Sciences, Beijing 100191, China

**Keywords:** Sjögren’s syndrome, immune infiltration, pro-inflammatory cytokine, epigenetic modification, IFN-related gene

## Abstract

Sjögren’s syndrome (SjS), characterized by keratoconjunctivitis sicca and dry mouth, is a common autoimmune disease, especially in middle-aged women. The immunopathogenesis of SjS is caused by the sequential infiltration of T and B cells into exocrine glands, including salivary and lacrimal glands. Effector cytokines produced by these immunocytes, such as interferons (IFNs), IL-17, IL-22, IL-21, IL-4, TNF-α, BAFF and APRIL, play critical roles in promoting autoimmune responses and inducing tissue damages. Epigenetic regulations, including DNA methylation, histone modification and non-coding RNAs, have recently been comprehensively studied during the activation of various immunocytes. The deficiency of key epigenetic enzymes usually leads to aberrant immune activation. Epigenetic modifications in T and B cells are usually found to be altered during the immunopathogenesis of SjS, and they are closely correlated with autoimmune responses. In particular, the important role of methylation in activating IFN pathways during SjS progression has been revealed. Thus, according to the involvement of epigenetic regulations in SjS, target therapies to reverse the altered epigenetic modifications in auto-responsive T and B cells are worthy of being considered as a potential therapeutic strategy for SjS.

## 1. Introduction

Sjögren’s syndrome (SjS) is the second most common chronic autoimmune rheumatic disease, characterized by dry eyes (sicca) and dry mouth. It is caused by abnormal infiltration and activation of immunocytes in the lacrimal glands (LGs) and salivary glands (SGs) due to multiple genetic and environmental factors. These infiltrated immunocytes lead to substantial progressive damage of the acinar structures responsible for saliva production and secretion. In addition, as indicated by some studies, the autoimmune responses may already occur before the immunocyte infiltration [1]. For example, Elke Theander et al. reported that anti-nuclear autoantibodies are present for up to 20 years before the diagnosis of SjS [2]. SjS occurs either alone, as primary Sjögren’s syndrome (pSS), or on the background of other autoimmune diseases as secondary Sjögren’s syndrome (sSS). Most SjS studies with patients or animal models are actually about pSS. Thus, in this paper, the SjS which we discuss also refers to pSS in principal and will be particularly mentioned as pSS if it was declared in the original study. The development of pSS can also cause extraglandular involvements of multiple organs, including fibromyalgia, arthralgia and interstitial nephritis, which suggests it is a systemic autoimmune disease [3]. In particular, patients with pSS exhibit an increased risk of non-Hodgkin B-cell lymphoma [4]. A recent study revealed that long before the clinical onset of the lymphoma, miRNA miR-200b-5p is found to be significantly downregulated in the minor SGs of pSS patients, indicating that this epigenetic regulation may be involved in the progression to non-Hodgkin B-cell lymphoma [5].

SjS generally develops in middle-aged women, but it can occur at any age throughout the population. Its prevalence is estimated to be around 3% in subjects aged 50 years and older, with a female to male ratio of 9:1 [6]. X-chromosome dosage effect is supposed to be associated with the high risk in females. In line with it, the disease risk increases 15 times in men with Klinefelter’s syndrome, which is genetically characterized by the presentation of one extra X chromosome (47, XXY) [7]. Similarly, the risk is also increased in women with trisomy X (47, XXX) [8]. In a recent study, X-linked genes in SG-derived mesenchymal stromal cells of pSS patients were found to exhibit miR-6891-5p-regulated skewing that is associated with alterations in H3K27me3 deposition. Thus, epigenetic regulations may be involved in the sexual bias of SjS; however, the precise mechanism still needs further study [9].

SjS is a multi-factorial disease caused by genetic, environmental and epigenetic abnormalities. The molecular etiology of autoimmune responses in SjS is still quite elusive. Genetic factors associated with SjS include particular HLA-DR allele subtypes and specific polymorphisms of genes, such as STAT4, IL-12A, TNIP1, IRF5 (type I interferon related), BAFF and BLK (related to B-cell activation) [10,11]. Environmental factors, such as infectious agents, are also considered as correlated with the pathogenesis of SjS. For instance, the Epstein–Barr virus (EBV) is implicated in the progression of SjS [12]. In addition, epigenetic regulations in the immune system, including DNA methylation, chromatin remodeling and non-coding RNAs, have been comprehensively studied in recent years, providing new insights into the pathogenesis and therapeutics of SjS. In addition, epigenetic changes are also involved in inflammatory responses in non-immune cells. It was reported that the DNA methylation process is abnormal in salivary gland epithelial cells (SGECs) from SjS patients [13,14]. Thus, given the potential role of epigenetic regulations in the auto-responsive immunocytes in SjS patients, in this review, epigenetic changes, particularly in the abnormally infiltrated and activated immunocytes in the exocrine glands, are particularly discussed.

## 2. Epigenetic Regulations in Immune Responses

**Figure 1:**
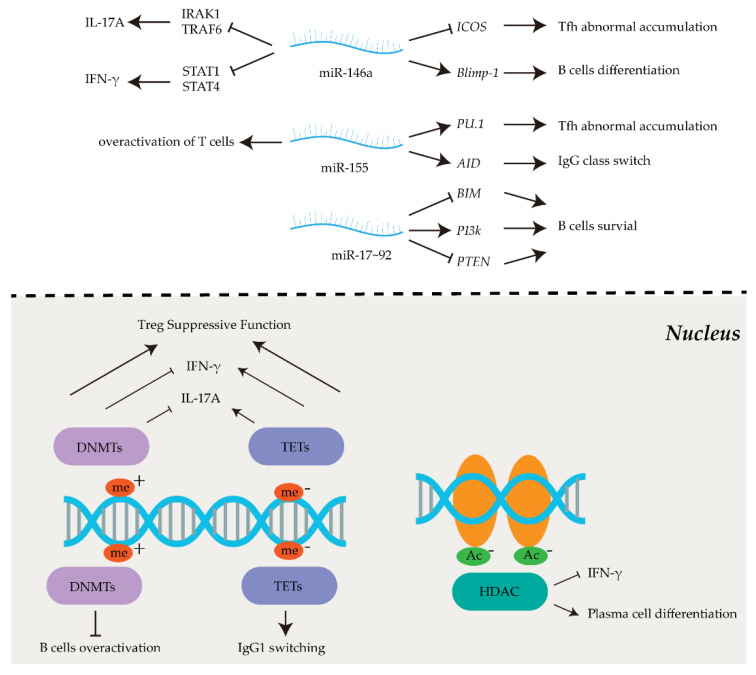
Epigenetic regulations in immune responses. Epigenetic modifications, such as DNA methylation (**bottom left**), histone modification (**bottom right**) and non−coding RNA expression (**top**), play critical roles in immune response. Abnormal epigenetic changes could lead to the dysregulation of immune responses.

Epigenetic modifications are mitotically and/or meiotically heritable and reversible alterations that induce upregulated or downregulated gene expression, without any changes in underlying DNA sequences. In addition, they also play a vital role in silencing or promoting the expression of non-coding sequences [15]. Epigenetic modifications are relatively stable over time, facilitating the maintenance of cell identity. However, they are also variable enough to change in response to various external and internal stimuli. Typical epigenetic modifications include methylation on DNA, modifications of histones, and the expression of non-coding RNAs (ncRNAs). They play an essential role in immune cell differentiation and activation [16]. Thus, their roles in regulating the aberrant differentiation and activation of immunocytes in autoimmune diseases have attracted much attention in recent years (Figure 1).

### 2.1. DNA Methylation

Methylation changes on DNA include both methylation and demethylation, which are, respectively, regulated by DNA methyltransferases, DNMT1, 3A and 3B, and Ten Eleven Translocation (TET)-1, 2 and 3 [17]. During DNA methylation, a methyl group donated by S-adenosyl-methionine (SAM) is transferred by DNMTs to the carbon-5 position of the cytosine pyrimidine ring (5mC) within a CpG dinucleotides. Methylated CpG sites then induce the structural changes of chromatin, making it difficult for transcriptional factor binding. CpG islands enriched in CpG base pairs are mainly distributed in the promoter and first exon regions of genes. Thus, DNA methylation in the CpG islands of chromatin is generally considered as a signal of gene silencing. Contrarily, the demethylation of DNA is the removal of a methyl group on cytosine through a serial process starting from the oxidation of 5mC to 5-hydroxymethylcytosine (5hmC) [17]. Additionally, demethylation, or 5hmC modification, of genes indicates enhanced gene expression.

DNA methylation and demethylation play critical roles in regulating the activation and differentiation of CD4^+^ T cells [18]. The most studied DNMTs in T cells are DNMT1 and DNMT3A. For example, during Th1 differentiation, both *Tbx21* and *Ifng* loci display demethylation and increased 5hmC level [16]. *Dnmt1* deficiency in T cells, or treating T cells with demethylation agents, results in enhanced Th1 effector cytokine expression [16]. DNMT3A is required to keep the *Ifng* locus stably silenced in Th17. The deletion of *Dnmt3a* results in demethylation at the *Ifng* locus of Th17 cells. Thus, under IL-12 stimulation, *Dnmt3a*-deficient Th17 become pathogenic and express higher levels of IFN-γ [19]. For Treg development and function, DNMT1, rather than DNMT3A, is essential. *Dnmt1*-deficient Treg lose their suppressive function both in vitro and in vivo [20]. Mice conditionally depleting *Dnmt1* in Treg die after 3 to 4 weeks due to severe systemic autoimmunity [20].

On the other hand, TET enzymes are also involved in T-cell differentiation. In Th1, T-bet recruits TET2 to the *Ifng* locus to keep it demethylated, while *Tet2* deficiency results in reduced IFN-γ expression [21]. In Th17, *Tet2* deficient leads to reduce 5hmc and RORγt binding at the *Il17a* locus, thus resulting in reduced IL-17 expression [21]. In Treg, Smad3 and STAT5 activated by IL-2 and TGF-β signaling recruit TET enzymes to the *Foxp3* locus, where they ensure the expression of Foxp3. Both TET1 and TET2 are required for the conversion of 5mC to 5hmC at the *Foxp3* locus, while their joint depletion results in impaired Treg differentiation and function [22]. TET enzymes display functional redundancy in Treg. Though depleting either *Tet2* or *Tet3* does not significantly impact Foxp3 expression, depleting them both leads to severely impaired Treg differentiation and stability [23]. TET expression and activity are enhanced by vitamin C and hydrogen sulfide in Treg. Thus, they are also capable of promoting the demethylation of conserved non-coding DNA sequence (CNS) elements at the *Foxp3* locus [22,24].

During Tfh differentiation, the involvement of TET and DNMT family members is still quite elusive. Nevertheless, in comparison with other Th cells, Tfh displays significantly reduced 5hmC at Bcl6 binding sites [18]. DNMT family members are found to be involved in B-cell activation and differentiation. The specific depletion of *Dnmt3a* and *Dnmt3b* in B cells does not affect their development and maturation; however, it results in the abnormal accumulation of plasma cells in the spleen and bone marrow. Compared to normal plasma cells, the *Dnmt3*-deficient plasma cells display reduced DNA methylation at over a thousand gene loci, and thus are unable to limit the activation and differentiation of B cells [25]. In addition, TET2 and TET3 also play a role in the class switch recombination of B cells. *Tet2* and *Tet3* deficiency in activated B cells results in substantially reduced 5hmC levels and defective IgG1 switching [26].

### 2.2. Histone Modifications

Histones play important regulatory roles in DNA replication and gene expression, highly relayed on their unique amino acid sequences. Histones are enriched with basic lysine and arginine residues, especially in the N-terminal tails, which are feasible for several post-translational modifications (PTMs), including methylation, acetylation, phosphorylation and ubiquitination [27]. Histone modifications stand for another type of important epigenetic regulation. These modified histones lead to altered chromatin structures or can act as binding sites for non-histone regulators, resulting in varied gene expression [17].

Multiple enzymes are involved in histone modifications, such as histone deacetylases (HDACs), histone acetyltransferases (HATs) and histone methyltransferase (HMTs). Several HDAC members have been reported to be involved in T- and B-cell responses [28]. For example, *Hdac1*-deficient Th1 display increased STAT1 phosphorylation and IFN-γ expression [29]. Additionally, *Hdac11*-deficient CD4^+^ T cells express higher Eomes and T-bet, and produce more IFN-γ [30]. Sirtuin 1(SIRT1), another member of HDAC family, shows higher expression in polarized Th17 than other Th effectors. It deacetylates RORγt in Th17, which then promotes its transcription regulator activity and enhances the effector function of Th17. SIRT1 inhibitor suppresses Th17 cells differentiation in vitro. Additionally, specifically depleting *Sirt1* in T cells protects mice from EAE, due to the reduced pathogenic differentiation of Th17 [31]. In addition, HDACs can interact with Foxp3 in Treg, and *Hdac6* or *Hdac9* deficiency leads to the increased suppressive function of Treg. As major microbial metabolites in the gut, short-chain fatty acids (SCFAs) can passively diffuse across the cell membrane of T cells and inhibit the activity of HDAC [32,33,34]. Studies in mice show that butyrate enhances H3 acetylation at the *Foxp3* locus of Treg by inhibiting HDAC, and thus promotes the transcription factor stability and activity [35]. In addition, butyrate activates mTOR and Blimp-1 in CD4^+^ T cells [36]. In addition to butyrate, the HDAC inhibitor valerate (VPA) also increases the activity of the mTOR complex and strongly induces IL-10 expression in T cells [37].

HDACs are also involved in regulating B-cell responses. The master regulator for plasma cell differentiation is Blimp-1. In undifferentiated B cells, Bcl-6 suppresses Blimp-1 expression by interacting with HDAC4, -5 or -7 and forming stable complexes [38]. When the HDACs are released from the complexes, histone acetylation at the promoter region of Prdm1 is increased, thus resulting in the upregulated expression of Blimp-1. In line with it, the HDAC inhibitor or butyrate is capable of inducing Blimp-1 expression in splenic B cells [39]. B cells were also reported to be regulated by SCFAs or HDAC inhibitors. One study showed that, in human and mouse B cells, butyrate, as well as the HDAC inhibitor VPA, upregulated miR-155, miR-181b and miR-361, which silenced *AICDA*/*Aicda* (encoding AID) mRNA, and upregulated miR-23b, miR-30a and miR-125b, which silenced *PRDM1*/*Prdm1* (encoding Blimp-1) mRNA [40]. Through these B-cell intrinsic epigenetic mechanisms, VPA decreased class-switched and hypermutated autoantibodies in lupus MRL/*Fas*^lpr/lpr^ mice, ameliorating the disease and extending mice survival. Thus, they also provide a therapeutic rationale and potential for other autoimmune diseases [40].

### 2.3. ncRNAs

Non-coding RNAs (ncRNAs) are single-strand RNA molecules transcribed from the genome. They do not encode proteins, but are implicated in cell development, proliferation and metabolism, playing important regulatory roles. Small non-coding RNAs (miRNAs) are 19–22 nucleotides in length, affecting gene expression at post-transcriptional levels, whereas long non-coding RNAs (lncRNAs) are defined as being above 200 nucleotides in length [17]. The miRNAs are novel molecular regulators of genes and pathways involved in immune responses in inflammatory and autoimmune diseases, such as RA, MS, SLE and SjS [41,42]. Under pathological conditions, the abnormal ex-pression of miRNAs leads to disease development by affecting the expression of multiple genes. About 2200 miRNAs have been found in the human genome [43]. Among them, about 50% are transcribed from non-coding regions, while the residues are distributed in introns. Each miRNA could target multiple genes, making them promising diagnostic biomarkers and therapeutic targets [44].

#### 2.3.1. Adaptive Immunity

There have been many studies showing the crucial role of miRNA in the development and differentiation of T and B cells [45,46]. One frequently reported miRNA in T and B cells is miR-146a. Mir146a deficiency increases T-cell reactivity and IL-17 secretion, through the reduction in the expression of TRAF6 and IRAK1 associated with the NF-κB signaling pathway [47]. Additionally, miR-146a regulates the expression of protein kinase C epsilon (PKCε), responsible for STAT4 phosphorylation and activation. Thus, miR-146a is also involved in inhibiting the Th1 differentiation-related pathway [48,49], whereas the deficiency of Mir146a in Treg results in an impaired immunological tolerance [50]. Additionally, miR-146a directly represses multiple messenger RNA (mRNA) targets, most prominently Icos. In line with it, Mir146a deficiency leads spontaneous Tfh accumulation [51]. In addition, miR-146a is also required for regulating the differentiation of B cells by promoting Blimp-1 expression [52,53,54].

There have been numerous studies indicating that certain miRNAs, such as miR-21, miR-27, miR-31, miR-138, miR-155, miR-146a, miR-181a and the miR-17~92 cluster, regulate T-cell development, differentiation and effector function [55,56]. Abnormal miRNA regulation can be detected in several autoimmune disease, including SjS [57]. MiR-146a has been reported to be upregulated in the PBMCs of SjS patients [58,59]. It serves as a negative regulator to inhibit the NF-κB signaling transducer TRAF6 (TNF-receptor-associated factor 6) and IRAK1 (IL-1-receptor-associated kinase 1) [60]. In line with it, miR-146a-deficient mice exhibit an abnormal activation of NF-κB signaling, leading to an overactivation of T cells [61,62,63]. In miR-146a-deficient mice, a deficiency of Treg was also found, correlated with the failure in suppression of Th1 responses [50]. In addition, miR-146a in Th1 and Th17 is also involved in regulating their differentiation, and thus is crucial for autoimmune responses [47,48,49]. Moreover, miR-146a negatively regulate the co-stimulatory signal of T cells by targeting Icos, thus limiting the accumulation of Tfh and GCs [51]. MiR-155 is another miRNA that is most commonly found to be dysregulated in the PBMCs of SjS patients [59]. The upregulated miR-155 is associated with the overactivation of T cells [59,64]. Additionally, the elevated expression of miR-181a is also found in the PBMCs of SjS patients [65]. MiR-181a participates in T-cell selection by dampening the TCR signal threshold against self-antigens, and thus the loss of miR-181a results in the development of autoreactive T cells [57].

Particular and abundant miRNAs are present at different stages of B-cell development. At an early stage, the miR-17∼92 cluster can promote the degradation of PTEN mRNA to increase PI3K activity, which further promotes B-cell survival [66]. In addition, the miR-17∼92 cluster regulates the survival of early B-cell progenitors by repressing the expression of the pro-apoptotic protein BIM [67]. In immature and transitional B cells, an abnormally increased miR-148a can suppress the expressions of Gadd45α, PTEN and BIM, which in turn leads to impaired B-cell tolerance [68]. The miR-17∼92 cluster is also involved in the c-Myc/miR-17∼92/PTEN axis to regulate the PI3K-mediated positive and negative selections of B cells [69]. At a mature stage, miR-155 can directly regulate PU.1, which is critical for the maintenance of germinal center (GC) responses [70]. Meanwhile, activation-induced cytidine deaminase (AID) is also a direct target of miR-155 in activated B cells, which is required for class switch recombination [71].

#### 2.3.2. Innate Immunity

MiRNAs also regulate the development and activation of innate immunocytes, such as ILCs and macrophages [72]. MiR-142 maintains ILC1 survival and function by promoting IL-15 signaling [73], whose deficiency results in reduced ILC1s in cancers [74]. MiR-142 and miR-146 are required for IL-5 and IL-13 secretion by ILC2, through upregulating ST2 [75,76]. The role of miRNAs in ILC3 is relatively unclear. A related study is limited to ILC3s in decidua, where miR-125 and miR-574 prevent the abnormal activation of ILC3 during pregnancy by inhibiting IL-6-STAT3 signaling [77]. In macrophages, their polarization towards M1 or M2 phenotypes plays important roles in diverse inflammatory responses [78]. MiRNAs are also found to affect polarization. MiR-9, miR-127, miR-155 and miR-125b promote M1 polarization, while miR-124, miR-223, miR-34a, let-7c, miR-132, miR-146a and miR-125a-5p promote M2 polarization [79]. The role of miR-21 in macrophage polarization is controversial. Some studies show that it promotes M1 polarization [80,81], but others suggest that it promotes M2 polarization [82,83]. Dendritic cells are also regulated by miRNAs. For instance, miR-21 promotes dendritic cell maturation, which thus helps to protect the kidney from ischemia-reperfusion injury [84].

## 3. Immunocyte Infiltration in SjS

**Figure 2:**
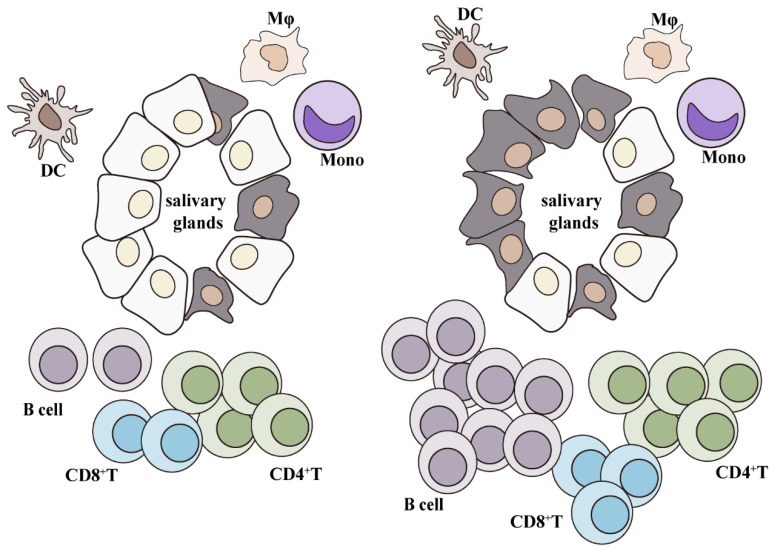
Graphics of immunocyte infiltration during the pathogenesis of SjS. At an early stage, the majority of infiltrated immunocytes in SGs of SjS patients are T cells (**left**), while, at a later stage, the prominent infiltrated immunocytes are B cells (**right**).

Though the precise mechanism for the pathogenesis of SjS remains largely unclear, the infiltration of immunocytes in exocrine glands is usually considered associated with disease progression. Immunocytes infiltrated in SGs of SjS patients mainly include T and B cells. At an early stage, CD4^+^ T cells constitute the predominant infiltration in SGs, while at a later stage, B cells are gradually accumulated [85,86]. This sequential infiltration and activation of CD4^+^ T and B cells are closely correlated with damages in the glandular tissues of SjS patients. In addition, CD8^+^ T cells are also crucial players in the immunopathogenesis of SjS, contributing to acinar injury. Overall, the infiltration of T and B cells are usually closely associated with SjS progression [87]. In addition, several recent studies have also revealed the infiltration and aberrant activation of other types of immunocytes during SjS. Thus, in this section, various infiltrated immunocytes in SjS are discussed (Figure 2).

### 3.1. T Cells

Infiltrated T cells exist throughout the progression of SjS and act as key drivers of the disease. The aberrant activation of T cells is then critical for the progression of the disease especially at an early stage, through recruiting other immunocytes, secreting pro-inflammatory cytokines and promoting B-cell activation and autoantibody secretion [88,89,90,91]. Their frequency gradually declines, but they persist throughout disease progression [92,93]. Thus, the frequency of effector T cells in severe lesions is usually lower than in mild lesions [85]. In addition, regulatory T cells (Treg) are also found to infiltrate in SjS lesions. Their frequency reaches a peak earlier than effecter T cells in intermediate lesions [94]. Though the frequency of T cells is inversely proportional to disease severity, the role of T cells on disease onset should not be ignored.

#### 3.1.1. Th1 and Th17

Type 1 and type 17 T-helper cells (Th1 and Th17), as major sources of IFN-γ and IL-17 in many autoimmune diseases [95,96,97,98], also play critical roles during the immunopathogenesis of SjS. In SjS patients, both IFN-γ and IL-17 are increased in T-cell-rich areas around SG ducts. In addition, Th1-cell-related (e.g., Ifng, Tnf and Tbx21) and Th17-cell-related (e.g., Il17, Il6 and Rorc) mRNA transcripts are also enriched in SGs and blood [88,89]. Early in the disease, Th1 cells infiltrate into SGs via the chemokine receptor CXCR3 [90]. The increment in IFN-γ+ cells in SGs is accompanied by a decrease in IFN-γ in the blood, suggesting that Th1 cells in blood circulation migrate to the SGs [99]. Th1 further trigger the infiltration of immune cells through secreting IFN-γ [90]. On the other hand, Th17 cells are the main pathogenic factor of SjS. They are increased in the peripheral blood of SjS patients with intermediate and severe lesions. IL-17A plays important roles in the progression of SjS, as indicated by the fact that IL-17 knockout mice are resistant to SjS disease models [100]. Moreover, IFN-γ+IL-17A+ Th17 cells usually exerting pathogenic roles in many autoimmune diseases also exist in the SGs of SjS patients [101]. However, whether the plasticity of Th17 cells plays an important role in disease progression still requires further investigation. Thus, in SjS patients, Th1 and Th17 may mainly exert their pathogenic roles by promoting immunocyte infiltration and inducing damages in their infiltrated glandular tissues, respectively.

#### 3.1.2. Tfh

B cells are gradually accumulated at a late stage of SjS [102]. T-follicular-helper cells (Tfh) play critical roles in helping B-cell responses [103]. They are hallmarked by the expression of CXC chemokine receptor 5 (CXCR5), inducible T-cell co-stimulator (ICOS) and programed death 1 (PD-1), and are regulated by master transcription factor B-cell lymphoma 6 (BCL-6) [104]. They promote B-cell activation, germinal center (GC) formation and antibody class switching [103]. Over the past decade, Tfh have been associated with a wide range of B-cell-mediated autoimmune diseases, including SjS [91]. The frequencies of Tfh cells are significantly increased in the glandular tissue and peripheral blood of SjS patients with mild lesions, and are positive correlated with disease severity [105]. Consistently, IL-6, which facilitates Tfh differentiation, is also elevated in the SGs and blood of SjS patients. However, whether Tfh are recruited into lesion tissue or differentiated within the tissue is still unclear. The increased Tfh play important roles in aggravating the disease. Tfh migrate to the B-cell zone in the ectopic germinal centers of salivary glands through the chemokine receptor CXCR5, where they promote GC B-cell differentiation, development and maturation by producing the effector cytokine IL-21 [106]. In addition, IL-4, contributing to GC formation in SjS patients, is also mainly secreted by the Tfh co-expressing transcription factor GATA3 [107]. Therefore, Tfh differentiation is closely correlated with autoimmune B-cell responses in SjS patients.

#### 3.1.3. Treg

Although Treg infiltration in the exocrine glands of SjS patients has been substantially studied, it is still unclear whether Treg play anti-inflammatory roles in the disease [108,109]. Several reasons, including states of the disease, markers for Treg identification as well as the balance between Treg and Th17, result in difficulties in clarifying the role of Treg in SjS [92,110]. For example, CD25 was generally used as a marker to identify Treg in SjS patients in many previous studies; however, it was also upregulated in activated CD4+ T-effector cells [111]. Treg exert their roles by inhibiting the activation of effector T cells. However, even when Treg frequency reaches a maximal level in SjS patients, the disease does not resolve, but rather it is usually aggravated, with the further increased frequency of effector T cells. The increment in Treg usually leads to a reduction in Th17-cell numbers; however, this negative correlation does not exist in SjS patients [110].

A particular subpopulation of Treg, follicular regulatory T cell (Tfr), is substantially increased in the SGs and blood of SjS patients [112,113]. These cells are distinguished by CD4^+^Foxp3^+^CXCR5^high^PD-1^high^Blimp-1^+^. They exert a suppressive role for Tfh proliferation and GC B-cell activation. The ratio of Tfr/Tfh in the blood of SjS patient is a biomarker for disease severity [112,113]. However, specific relationship between the Tfr/Tfh ratio and B-cell hyperactivation in SjS patients is still unclear. Tfr are also capable of downregulating B7-1 or B7-2 expression on B cells in GC, thereby potentially alleviating autoimmune responses and the severity of inflammation [114]. In general, comprehensive studies about the changes in Treg of SjS patients are still needed to further understand their roles in disease progression.

#### 3.1.4. CD8^+^ T Cells

CD8^+^ T cells exert pathogenic roles by secreting cytotoxic molecules, such as granzyme B (GzmB) and perforin, and inflammatory cytokines, including interferon-γ (IFN-γ) and tumor necrosis factor-α (TNF-α) [115]. Recent studies have revealed that CD8^+^ T cells are enriched around the apoptotic acinar epithelial cells of SjS patients. IFN-γ in SGs is involved in the recruitment of more CD8^+^ T cells. *Ifng* deficiency was reported to abrogate CD8^+^ T-cell infiltration and decrease gland destruction in SjS mice. CD8^+^ T cells infiltrated in SGs displayed a tissue-resident phenotype CD69^+^CD103^+/−^ and exhibited dramatically increased IFN-γ production. In a *p40*^−/−^*Il2ra*^−/−^ murine model of SjS, CD8^+^ T-cell depletion, via either genetic *Cd8a* deficiency or antibody-mediated depletion, fully protected the mice from pathologic manifestation [116]. Moreover, even after the onset of the disease, antibody-mediated CD8^+^ T-cell depletion successfully restored the secretory function of SGs, suggesting it as a potential therapeutic strategy. Thus, the correlation between CD8^+^ T cells and gland destruction in SjS patients should receive more attention in the future.

### 3.2. B Cells

B cells are central to the pathophysiology of SjS at a late stage [102]. In SjS patients, B-cell subpopulations in circulation are disturbed, and the frequency of GC founder cells is usually increased. This B-cell subpopulation expresses high levels of CD19 and tyrosine-protein kinase BTK, which together increase BCR signals [117,118]. The aberrant activation of B cells also increases the risk of SjS patients to develop B-cell lymphoma, such as non-Hodgkin’s B-cell lymphoma [119,120].

B cells promote the progression of autoimmune diseases mainly through their roles as cytokine producers, antigen-presenting cells or autoantibody secretors. B cells possess the capacity to produce a range of cytokines, including either the pro-inflammatory cytokines IL-6 and TNF-α or the anti-inflammatory cytokines TGF-β and IL-10 [117]. The difference is relayed on the polarization of B cells under different circumstances [117]. However, which kind of cytokine is mainly secreted by B cells in SjS patients has not been clearly reported. In the SGs of SjS patients, B cells also act as antigen-presenting cells, activating naive T cells in an MHC-II-dependent manner and, thereby, promoting disease progression [121].

A key feature of SjS is that target organs, including salivary and lachrymal glands, are involved in B-cell activation, especially the formation of GC-like structures within the epithelium and plasma cell niches [122]. Thus, in these organs, autoantibodies produced by autoreactive B cells are significantly enhanced. For example, the production of many autoantibodies, such as anti-salivary gland protein 1, anti-carbonic anhydrase 6 and anti-parotid secretory protein, are increased in pSS patients [123]. These autoantibodies participate in immune complex formation, which is a vital step in the progression of SjS.

### 3.3. Innate Lymphoid Cells

Innate lymphoid cells (ILCs) are tissue-resident innate lymphocytes that play crucial roles in regulating tissue homeostasis [124]. Unlike T and B cells, they do not express antigen specific receptors. According to their difference in effector functions, ILCs are divided into three subsets, ILC1s, ILC2s and ILC3s, mirroring the CD4^+^ Th subsets Th1, Th2 and Th17 [124]. The discovery and investigation of ILCs over the past decade have been closely associated with autoimmune disease [125]. For example, increased Fas expression on ILC2s and ILC3s decreases their frequency in the blood of SLE and pSS patients, potentially altering the homeostasis of ILCs [116]. ILC3 is the main source of IL-22, which is supposed to play a pathogenic role in epithelial damage in SjS patients [126].

### 3.4. Antigen-Presenting Cells

In addition to B cells, other antigen-presenting cells, such as DCs and macrophages, are also increased in the SGs of SjS patients [127,128]. Both DCs and macrophages are differentiated from monocytes in the blood, possessing a high antigen-presenting capacity. They are positively correlated with the severity of SjS [127]. In addition, SGECs also play an antigen-presenting role, by upregulating MHC molecules during the onset of SjS [128]. Thus, these antigen-presenting cells are indispensable for the aggregation and activation of inflammatory cells in SjS patients.

## 4. Pro-Inflammatory Cytokines in SjS

As previously mentioned, the immunopathogenesis of SjS is initiated by Th1 and Th17, and progressed by Th2 and Tfh, which then ignite B-cell responses, resulting in more severe tissue lesions [129]. In line with it, cytokines acting on or produced by these immunocytes, including interferons (IFNs), interleukins and tumor-necrosis-factor (TNF) superfamily members [130], play critical roles in disease progression.

**Figure 3:**
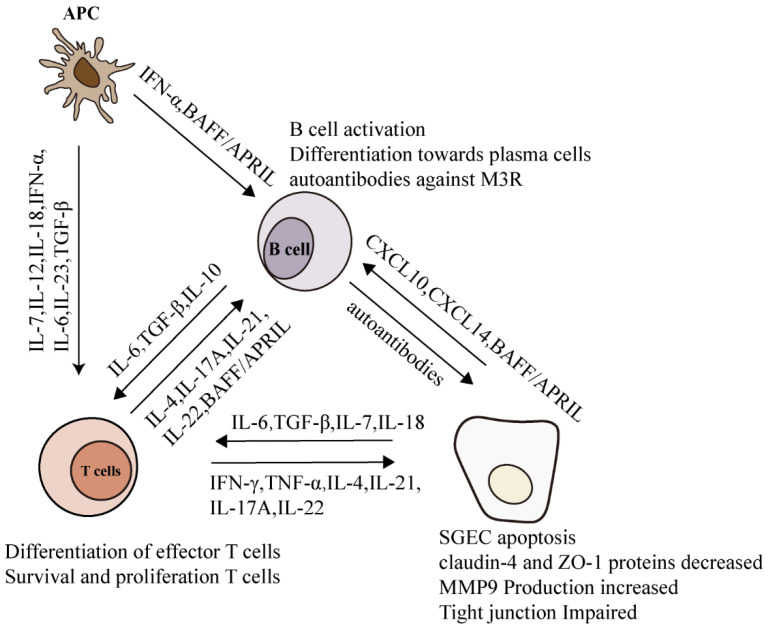
Mutual interactions between T cells, B cells and SGECs result in the immunopathogenesis of SjS. Infiltrated T and B cells in the SGs of SjS patients produce pro-inflammatory cytokines, such as IFNs and interleukins, acting on them mutually to further aggravate the autoimmune response. In addition, these pro-inflammatory cytokines also act on SGECs, inducing the destructions of glandular tissues.

After processing certain autoantigens, antigen-presenting cells are activated to produce IL-7, IL-12, IL-18 and type I IFNs, which effectively promote Th1 responses [131]. IFN-γ and TNF-α produced by Th1 cells then induce CXCL9/10 secretion from the epithelial cells and stromal cells of SGs [131]. Additionally, the activated antigen-presenting cells produce cytokines required for Th17 cell differentiation, including IL-6, IL-23, TGF-β and IL-1β. Differentiated Th17 release effector cytokines, such as IL-17, IL-22 and IL-21 [101]. IL-21, also released by Tfh cells, facilitates the formation of GC-like structures within epithelium and plasma cell niches [132]. IL-21 and IL-17 derived from Tfh or Th17, together with IL-4 from Tfh and Th2, promote B-cell proliferation, differentiation and autoantibody secretion [133]. In addition, the TNF superfamily cytokines BAFF (B-cell-activating factor) and APRIL (a proliferation-inducing ligand) are also involved in promoting B-cell responses [134]. In this section, the crucial cytokines and their effector roles during SjS are summarized (Figure 3).

### 4.1. Interferons

High levels of IFNs and the elevated transcription of IFN-stimulated genes (ISGs) are found in the SGs and serum of SjS patients, as well as SjS animal models, indicating an important role of IFNs in the pathogenesis of SjS [47,48,49,50]. Interferons are classified into three types, type I interferons (IFN-α and IFN-β), type II interferon (IFN-γ) and type III interferon (IFN-λ) [135,136].

#### 4.1.1. Type I interferons

In the inflamed SGs, IFN-α are mainly produced by plasmacytoid dendritic cells (pDCs), after they recognize autoantigens through the Toll-like receptors (TLRs) TLR-7 and TLR-9 [137,138]. On the other hand, IFN-β is produced by SGECs, also through TLR signaling [139,140]. Once engaged with their receptor (IFNAR) on the target cells, type I IFNs initiate the transcription of ISGs [141,142]. ISGs are generally considered to execute antiviral and antitumor activities [143]. However, they also contribute to autoimmune responses in SjS, through triggering the secretion of pro-inflammatory cytokines and chemokines, such as CXCL10 and BAFF, class switch of immunoglobulins and cellular cytotoxicity of NK and T cells [90,144,145,146]. In line with this, mice lacking IFN-α receptor 1 (*Ifnar1*^−/−^) display significantly reduced susceptibility to SjS-like disorders [147,148].

#### 4.1.2. Type II interferon

In addition to type I IFNs, type II IFN IFN-γ also play a crucial role in inducing tissue lesions in SjS patients. Thus, the lesion tissue is usually characterized by both type I and type II IFN signatures [149,150]. In SGs, IFN-γ is mainly generated by Th1 and Tfh. It also induces a subsequent upregulation of ISGs in either immune or non-immune cells [110,151]. This process promotes a further differentiation of naïve CD4^+^ T cells towards Th1 [152,153]. In addition, it also polarizes macrophages to an M1 phenotype, which usually correlates with tissue inflammation [152,153], whereas for non-immune cells, such as SGECs, IFN-γ upregulates their expression of MHC class II and co-stimulatory molecules (CD80, CD86 and CD40), which facilitate antigen presentation for adaptive immune responses [154,155,156]. In addition, IFN-γ induces epithelial damages through Fas-mediated apoptosis in SGECs and reduces epithelial integrity by disrupting tight junction (TJ) structures [157,158]. Moreover, as mentioned above, IFN-γ also facilitates the recruitment of more pro-inflammatory immunocytes.

#### 4.1.3. Type III interferon

Type III IFN, IFN-λ, shows a certain functional similarity to type I IFNs [136]. However, unlike the extensive study of type I and type II IFNs, type III IFN is the latest member added to IFN family and its role during inflammatory responses still requires further studies. As recently reported, IFN-λ is highly expressed by the ductal epithelium of SjS patients, which acts synergistically with type I IFNs to further upregulate the expressions of CXCL10 and BAFF [159].

### 4.2. Interleukins

The inflammatory milieu in SjS patients is characterized by a dysregulated cytokine network, especially pro-inflammatory interleukin overexpression. The major pro-inflammatory interleukins include IL-17, IL-22, IL-21 and IL-4, and exhibit aberrant upregulation in the SGs or serum of SjS patients [122,160].

#### 4.2.1. IL-17 and IL-22

Pro-inflammatory cytokine IL-17A is mainly expressed by Th17 cells in the inflamed SGs of SjS patients, and considerable evidence suggests IL-17A as a pathogenic factor in SjS [101]. The level of IL-17A in SG biopsies or serum is positively correlated with histopathological score of SjS patients [161,162]. It binds to its receptors on epithelial and stromal cells, and affects downstream gene expression [163,164]. Elevated IL-17A results in the significant downregulation of the TJ proteins laudin-4 and zonula occludens-I (ZO-1), impairing epithelial integrity and leading to SG dysfunction [165]. In addition, IL-17A is found to induce matrix metalloproteinase 9 (MMP-9) secretion by epithelial cells, which is also associated with the tissue damage [166,167]. In line with the crucial role of IL-17A in SjS pathogenesis, *Il17a*-deficient mice fail to develop SjS [100], while mice with overexpressed IL-17A in SGs exhibit a SjS-like phenotype [168].

IL-22 is another pro-inflammatory cytokine released by Th17 [101,169]. IL-22 is also found to be highly expressed in the SGs of SjS patients, correlating with their clinical manifestations [170]. IL-22 acts on epithelial and myeloid cells, and induces them to secret cytokines and chemokines, such as CXCL13 [170]. The formation of ectopic GC in the SGs of SjS patients is facilitated by IL-22 in a CXCL13-dependent manner [171]. In addition, IL-22, along with IL-17A, promote B-cell infiltration and Th17 polarization [126,170,171]. Deficiency of *Il22* reduces the infiltration of B cells in the SGs of SjS mice [171].

#### 4.2.2. IL-21

Accumulating evidence suggests that IL-21 plays a critical pathogenetic role in SjS. High levels of IL-21 and IL-21R are detected in the SGs and serum of SjS patients [172]. Additionally, several SjS-associated genes identified by genome-wide association studies (GWAS) are closely related to the IL-21 pathway [173]. IL-21-producing cells consist of Th17, Tfh and CCR9^+^ Th cells [174,175]. IL-21 displays a pleiotropic impact on the proliferation, apoptosis and differentiation of T and B cells, correlating with disease progression [173]. In addition, IL-21 promotes the proliferation and cytotoxicity of CD8^+^ T cells, which induces damages of SGECs [176]. Consistently, the local suppression of IL-21 in submandibular glands retards SjS-like symptoms in mice [177].

#### 4.2.3. IL-4

IL-4 level is found to increase in SGs of SjS patients, especially those exhibiting significant B-cell infiltration [129]. IL-4/STAT6 signaling pathway is essential in the progression of SjS-like diseases [178]. The major source of IL-4 in SjS patients is Tfh, rather than Th2 cells [107]. IL-4 promotes CXCL12 production from stromal precursor cells [179]. Additionally, IL-4, together with CXCL12, participate in regulating B-cell activation, migration and maturation [179,180,181]. The genetic deficiency of *Il4* or *Stat6* impedes antibody switches to IgG1 isotype against muscarinic acetylcholine type-3 receptor (anti-M3R) [182]. In addition, IL-4 also exacerbates inflammation in SjS patients by inducing the apoptosis of SGECs [183].

### 4.3. Tumor-Necrosis-Factor Superfamily Members

The TNF superfamily is composed of more than 20 structurally related protein ligands [184]. They exert important immune regulatory roles and are implicated in various autoimmune diseases [185,186]. In SjS patients, TNF superfamily members, such as TNF-α, BAFF and APRIL, are also upregulated, and play critical roles in the immunopathogenesis of the disease [187,188,189,190].

#### 4.3.1. TNF-α

TNF-α is involved in numerous autoimmune diseases, while anti-TNF-α treatment has also showed great promises as a therapeutic strategy [191]. In SjS patients, elevated expression of TNF-α is detected in both SGs and serum [189,190]. TNF-α is produced by many cells, especially Th1 and SGECs [190,192]. Alone, or in combination with IFN-γ, it induces the apoptosis of SG cells, which leads to the dysfunction of the tissue [193].

#### 4.3.2. BAFF and APRIL

BAFF and APRIL share similar biological roles in promoting B-cell differentiation, survival and autoantibody secretion [194,195]. The levels of BAFF and APRIL in serum positively correlate with the disease severity of SjS [188]. BAFF is derived by several types of cells, including antigen-presenting cells, T cells, neutrophils and SGECs, after they are activated by type I or type II IFNs [146,187]. Given its role in promoting B-cell responses, excessive BAFF results in the accumulation of autoreactive B cells and thus the disease progresses [146,187,196,197]. In addition, BAFF-transgenic mice also exhibit enhanced B-cell infiltration and aggravated hyperplasia in SGs. Furthermore, once they age, they mostly develop SjS-like diseases [198].

## 5. Epigenetic Modifications in SjS

**Figure 4:**
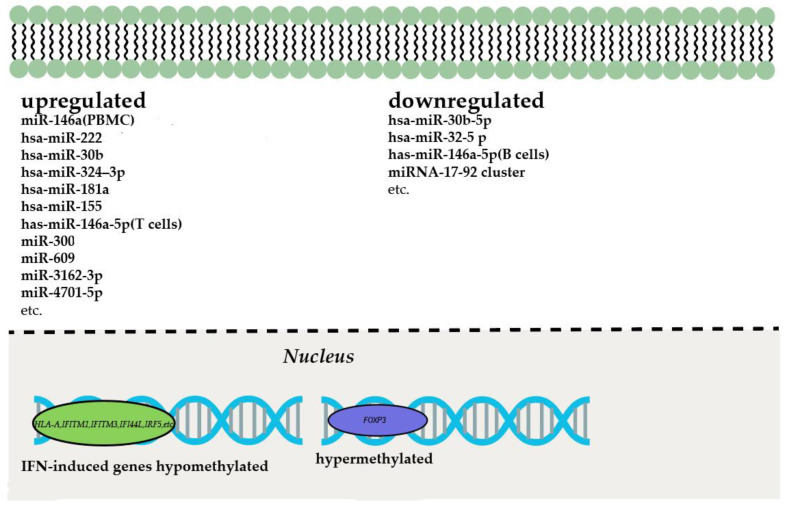
Epigenetic modification changes in immunocytes during SjS. IFN-related genes exhibit hypomethylation in the T and B cells of SjS patients. In addition, multiple miRNAs, correlated with IFN-related genes, are also altered in T and B cells, whereas the *FOXP3* locus in Treg is hypermethylated.

According to the role of epigenetic regulations in immunocyte activation, epigenetic changes can also be involved in the immunopathogenesis of SjS. Indeed, DNA methylation, histone modifications, as well as ncRNAs play important roles in the progression of SjS. They are involved in regulating the aberrant infiltration and activation of T and B cells and promote the production of pro-inflammatory cytokines during SjS immunopathogenesis (Figure 4).

### 5.1. Epigenetic Modification of IFN-Related Genes

Numerous studies have confirmed the important role of the IFN pathway in the immunopathogenesis of SjS. In pSS patients, disease progression can be divided into three key stages: activation of the innate immune system, especially via the IFN pathway; the activation of T and NK cells due to HLA-related predisposition and through IL-12–IFN-γ pathways; and the activation of B cells, presumably through CXCR5-mediated recruitment to lymphoid follicles and B-cell receptor activation. Epigenetic modifications are critical in activating the IFN pathway [199].

The correlation between DNA methylation and disease progression in pSS patients has been substantially studied. DNA methylation was compared between the naïve CD4^+^ T cells of 11 pSS patients and 11 healthy cohorts in an epigenome-wide study [13]. The hypomethylated CpG sites in pSS patients are related to genes of the IFN pathway, such as *STAT1*, *IFI44L*, *USP18* and *IFITM1*. In another comprehensive study of DNA methylation of whole blood, CD19^+^ B cells as well as minor SG biopsies from pSS patients, prominent hypomethylation on IFN-regulated genes was also confirmed, which correlated with their increased expression. Furthermore, the top 12 differentially methylated CpG sites in pSS patients, such as *MX1*, *IFI44L*, *PARP9*, *PLSCR1*, *IFIT1*, *IFITM1* and *HLA-A*, were found to be mainly influenced by the presence of anti-Sjögren’s Syndrome antigen A (SSA) and/or anti-Sjögren’s Syndrome antigen B (SSB) antibodies [13,200]. The genome-wide comparison of DNA methylation profiles in the CD4^+^ T cells and CD19^+^ B cells of pSS patients and age-matched health cohorts using 450K BeadChip suggested that the major methylation alterations were present in B cells and genetic at-risk loci [199]. The DNA methylation changes in B cells were further quantified by Pyrosequencing, and alterations in IFN-related genes, such as *IFITM1*, *IFITM3*, *IFI44L* and *IRF5*, were validated. Intriguingly, the methylation status of B cells was closely correlated with disease progression. Differentially methylated genes in patients positive for at least one pSS-specific autoantibody (anti-SSA, anti-SSB or anti-SSA/anti-SSB) showed a significantly enriched IFN signature [199].

Histone modifications in SjS patients are still quite elusive, while expression changes on miRNAs are reported in some studies. The differential expression of miRNAs, including hsa-miR-378a-3p, hsa-miR-222-3p, hsa-miR-26a-5p, hsa-miR-30b-5p and hsa-miR-19b-3p, were discovered in a comparative study of CD4^+^ T cells and CD19^+^ B cells from pSS patients and healthy cohorts [11,201]. Of them, hsa-miR-30b-5p is one of the most differentially expressed miRNAs in the SGs of pSS patients [202]. Additionally, hsa-miR-146a-5p is known to aggravate inflammatory responses. It targets IRF5, STAT1 and IRAK1, which exert regulatory roles in the IFN and NF-kB pathways [61].

### 5.2. Epigenetic Modifications in B Cells

As mentioned above, in SjS patients, methylation changes in B cells are more obvious than in T cells, suggesting an important role of B cells in SjS progression. The hypomethylation of IFN-induced genes, including STAT1, IFI44L and IFITM1, was found in peripheral blood B cells, especially from SSA and/or SSB antibody-positive patients [200]. The hypermethylated sites in the B and T cells of SjS patients overlap with histone modifications, corroborating that the related transcriptions are inactivated, whereas hypomethylated sites usually overlap with enhancer sites [203].

SjS patients often exhibit increased SSA and SSB autoantigens, correlated with the aberrant expression of the miRNAs targeting them. In minor SGs and peripheral blood of SjS patients, a large number of miRNAs display differential expression patterns. The miRNA difference also exists in T and B cells from the peripheral blood of SjS patients, especially those positive for anti-SSA autoantibodies. The expression of 372 miRNAs in purified blood cells from SjS patients and health cohorts were analyzed in a recent study. A total of 21 miRNAs showed significant expression differences in T cells, while 24 differentially expressed miRNAs were identified in B cells [201]. Among the differentially expressed miRNAs, hsa-miR-30b-5p was previously reported to negatively regulate BAFF expression. Thus, it may play a role in regulating B-cell function [202].

### 5.3. Epigenetic Modification of Foxp3

Treg exerting immune suppressive functions are quantitatively and qualitatively defective in SjS patients. Foxp3 (forkhead box p3) is the master transcription factor of Treg, which dictates their development and function [204]. Its expression is substantially regulated by epigenetic modifications. For example, the methylation status of the FOXP3 promoter region determines its expression. In a study of 15 pSS patients, the FOXP3 promoter in CD4+ T cells was found to be hypermethylated, correlated with the significantly reduced expression of FOXP3 mRNA and protein [205].

### 5.4. Correlation between SjS-Related SNPs and Epigenetic Changes

GWAS has been used to assess the association of genetic variation and unique traits in SjS patients, and an increasing number of susceptible SNPs have been identified [11,206,207,208]. Though genes that directly induce SjS have already been identified through the large studies [10,11], several SNPs in molecules of signaling pathways, including IFN signature (IRF5 STAT1, STAT4 and IL12A), B- and T-cell signaling (BAFF, GTF2I, TNFSF4, CXCR5, CCL11 and TNFAIP3) and the NF-κB pathway (TNIP1, CARD8, IKBKE, IRAK1 and TANK), do appear to be involved in the pathogenesis of SjS. Given the fact that most SNPs are located in noncoding sequences, they are supposed to potentially affect the regulation of genes, resulting in altered transcription levels, splicing manners and epigenetic modifications [209]. In a GWAS performed to discover the difference between pSS patients and healthy controls, a SNP in the methyl-CpG-binding protein 2 (MECP2) gene (rs17435) on the Χ chromosome was identified, which, for the first time, verified the genetic association between SNPs on the Χ chromosome and pSS [210]. MECP2 is associated with DNA methylation-induced transcriptional silencing. In SLE, it has been reported to be associated with disease susceptibility [211]. Accordingly, this SNP may be also involved in the pathogenesis of pSS. Nevertheless, further studies are still needed to unravel the correlation between SjS-related SNPs and the aberrant epigenetic modification changes during the pathogenesis of SjS.

## 6. Epigenetic Therapeutics of SjS

SjS manifests in various ways, typically characterized by keratoconjunctivitis sicca and dry mouth. Fatigue infections also usually threaten the patients. Thus, the major therapeutic aim for most SjS patients is to improve the quality of their lives by ameliorating the sicca and fatigue symptoms. Anti-inflammatory treatments, such as topical corticosteroids, topical cyclosporine and oral secretagogues, are commonly implied to prevent the pathogenesis of the disease [157]. In addition, targeting therapy, such as Rituximab, a monoclonal anti-CD20 antibody for depleting auto-responsive B cells, has also shown promising therapeutic results. The efficacy of rituximab in reducing fatigue in pSS patients was studied in a double-blind, placebo-controlled randomized trial, which showed significant improvement in the rituximab group [158].

The cellular and molecular etiology of SjS is quite complicated, considering the immunocytes and effector cytokines involved in the autoimmune responses. In addition, they are also dynamically changing along with the immunopathogenesis of the disease. Thus, new therapies targeting pro-inflammatory cytokines or autoreactive immunocytes in SjS patients are still worth exploring. Based on our description, epigenetics is intensively involved in the pathogenesis of SjS, and it shows broad impacts on the abnormal activation of T and B cells, as well as their aberrant production of pro-inflammatory cytokines. Accordingly, we particularly considered the possibility of taking epigenetic targets as a strategy for SjS therapy. Actually, epigenetic drugs have already been used in other autoimmune diseases, especially RA and SLE. Increased DNA methylation in CD4^+^ T cells is also found in RA, associated with their autoimmune responses. Methotrexate (MTX), a first-line drug for RA treatment through antagonizing folate metabolism, affects one-carbon metabolism to disrupt the methyl transfer process of CpG methylation [212]. In addition, the inhibition of histone methyltransferase Ezh2 is currently under evaluation for the therapy of SLE. Ezh2 inhibition by DZNep significantly reduces renal inflammation and improves the survival of MRL/lpr spontaneous lupus mice before and after the disease onset [135]. Additionally, a preliminary study demonstrated that the HDAC inhibitors VPA and butyrate diminished plasma cell differentiation in MRL/lpr spontaneous lupus mice, with unaffected B-cell viability and proliferation [40].

Given the involvement of epigenetic regulators, such as DNMT1, DNMT3A, DNMT3B, TET2, TET3, HDAC1, HDAC11, SIRT1 and miR-146a, in SjS immunopathogenesis, they can all be possibly considered as therapeutic targets. In particular, as previously mentioned, the IFN pathway plays critical roles in promoting SjS progression, while it is well studied that methylation changes in T and B cells are closely associated with the activation of IFN-related genes. Thus, perhaps targeting therapies towards the abnormal methylation changes in the auto-responsive immunocytes of SjS patients should be particularly considered in future.

## 7. Conclusions

SjS is a common chronic autoimmune rheumatic disease, caused by complicated cellular and molecular etiologies. Disease progression is companied by the abnormal activation of T and B cells sequentially, including Th1, Th17, Tfh and autoreactive B cells. Key effector cytokines produced by these immunocytes are closely correlated with the autoimmune responses and the damages induced in glandular tissues. The involvement of epigenetic regulations during immune cell activation has attracted considerable attention and has been intensively studied in the past. In particular, epigenetic changes in auto-responsive immunocytes from SjS patients have also been discovered. Thus, unraveling the involvement of epigenetic changes in SjS progression has provided many new insights into its immunopathogenesis and therapy. Especially, these studies reveal that IFN-related genes are hypomethylation in multiple immunocytes, such as T and B cells, as well as SGECs, which play critical roles in the immunopathogenesis of SjS. These discoveries suggest that epigenetic targeting could be considered as a potential therapeutic strategy of SjS. The application of epigenetic treatment in other autoimmune diseases, such as RA and SLE, further demonstrates this possibility. Nevertheless, more studies about the epigenetic modification changes and their impacts on pro-inflammatory cytokine production and SjS progression are still required to precisely design and adjust the epigenetic therapy.
